# Depression as a non-causal variable risk marker in coronary heart disease

**DOI:** 10.1186/1741-7015-11-130

**Published:** 2013-05-15

**Authors:** Anna Meijer, Marij Zuidersma, Peter de Jonge

**Affiliations:** 1 Interdisciplinary Center Psychopathology and Emotion Regulation, University of Groningen, University Medical Center Groningen, Hanzeplein 1, Groningen, 9713 GZ, The Netherlands

**Keywords:** Cardiovascular disease, Depression, Causality, Risk marker, Risk factor

## Abstract

**Background:**

After decades of investigations, explanations for the prospective association between depression and coronary heart disease (CHD) are still incomplete.

**Discussion:**

Depression is often suggested to be causally related to CHD. Based on the available literature, we would rather argue that depression can best be regarded as a variable risk marker, that is, a variable that fluctuates together with mechanisms leading to poor cardiovascular fitness. Despite numerous efforts, no evidence is found that manipulation of depression alters cardiovascular outcomes - a key premise for determining causality. To explain the concept of a variable risk marker, we discuss several studies on the heterogeneity of depression suggesting that depression is particularly harmful for the course of cardiovascular disease when it appears to be a physiological consequence of the cardiovascular disease itself.

**Summary:**

We conclude that instead of depression being a causal risk factor for CHD, the association between depression and CHD is likely confounded, at least by the cardiac disease itself.

## Background

For many decades, researchers and clinicians have observed an association between depression and coronary heart disease (CHD), and have tried to discover the mechanisms involved. A fair number of theories have been formulated and investigated, but no conclusive explanation exists as to why depression might lead to CHD and its progression. One potential explanation is that depression is causally related to the development and clinical course of CHD, either directly or through mediating pathways. Suggestive of causality, the association between depression and CHD has been consistently found across different settings and patient groups, as both etiological and prognostic associations between depression and CHD have been found in various studies of research groups worldwide [1-4]. However, in the present review we will argue that, instead of being causally involved, depression can best be defined as a variable risk marker of CHD and its progression. We will discuss this on the basis of Hill’s criteria for causality [5]. In addition, we will discuss current evidence in the light of the idea that symptoms and subtypes of depression that appear to be a physical consequence of the cardiac disease are most strongly predictive of adverse cardiovascular outcomes.

Principles for establishing causality have been developed by Bradford Hill [5] (Table 1), which can aid in determining the plausibility of a causal association between two variables. Below, we will discuss how the principles most relevant for the association between depression and CHD (indicated with an *), substantiate the hypothesis that depression is a variable risk marker for CHD.

**Table 1:** **Principles of causality**[5]

**Principle**	**Explanation**
Strength	the stronger the association, the more likely is causality*
Consistency	the association exists in different contexts and times
Specificity	the association is specific for the variable and one particular outcome*
Temporality	the variable precedes the outcome*
Dose–response relationship	an increase in the variable results in an increase in the outcome*
Plausibility	plausible theories/mechanisms for explaining the association exist*
Coherence	the causal theory is coherent with existing knowledge
Experimental manipulation	manipulation of the variable results in changes in the outcome*
Analogy	similar associations exist between different variables

Principles marked with an * are the principles discussed in the text.

The *principle of strength* states that causality is more likely in stronger associations. The association between depression and CHD, however, is generally moderate. Otherwise healthy people with depression have an 80% higher risk of dying of CHD or having a myocardial infarction (MI) than those without a depression [2]. Similarly, depressed patients with established CHD have a two times higher risk of dying of CHD or other causes [1,2]. Although the association of depression with development and progression of CHD is generally moderate, its magnitude is similar to that of the associations of other well-known risk factors for CHD, including smoking, hypertension, diabetes, obesity, dietary pattern, alcohol consumption and physical activity [6]. The causality of each of these factors (including depression), as well as their place in the causal chain towards CHD, remains to be resolved. However, the absence of a strong association suggests the association is not definitely causal. Although causality is not excluded as a possibility, it is also possible that [7,8] other factors, such as cardiac disease severity and other health-related risk factors explain at least part of the association between depression and CHD.

The *principle of specificity* states that associations are more likely to be causal when they are specific for one variable and one outcome. According to Hill the argument of specificity is difficult to attain, and can only argue strongly in favor of causality, but can never argue against it [5]. If an association is very specific (that is, one risk factor associated with one disease only), then this is strongly supportive for causality. However, when an association is not specific, this does not necessarily argue against causality. For instance, smoking is associated with many disease outcomes, but may still be causally related to the development of some of the diseases. Like smoking, depression is associated with the outcomes of multiple diseases [9-14], and etiologically, depression is a risk factor for the development and progression of several types of disease [10], not just CHD. Moreover, other psychological problems have also been found to be associated with the development and progression of CHD, such as anxiety [15,16], vital exhaustion [17,18], anger and hostility [19]. Therefore, Hill’s principle of specificity has not been met, meaning that there is no support that depression is definitely a causal risk factor.

The *principle of temporality* states that, in a causal association, the determinant variable must precede the outcome. This principle means to determine the absence of causality rather than its presence. That is, an association in which the determinant does not precede the outcome is by definition not causal, but associations in which the determinant does precede the outcome may still be confounded. In the association between depression and CHD, most of the evidence points towards a bidirectional role of depression in CHD. In one direction, depression indeed precedes CHD, as depression in otherwise healthy people is associated with increased risk for the onset of CHD [2,20-23], with a pooled risk ratio (RR) of between 1.5 and 2.0 [2,21,22]. In addition, in patients with existing CHD, depression is associated with an increased risk of cardiac morbidity and (cardiac) mortality [1,2,24].

In the other direction, CHD is a potential risk factor for depression, as CHD is associated with increased prevalence of depression. An estimated 17% to 27% of CHD patients have major depressive disorder (MDD), compared with around 5% in the general population [25,26]. In addition, CHD in older persons without depression at baseline was found to be associated with an increased risk of developing depressive symptoms two years later [27]. Furthermore, a recent study, using the largest sample (n = 1,117,292) to date, found cardiovascular fitness in healthy young men to predict depression up to 40 years later, even without cardiac disease being present [28]. This latter study suggests that poor cardiovascular fitness causes depression later in life, but may also mean that both depression and CHD are caused by other factors, such as family environment and inactivity. Thus, the principle of temporality has been met as depression precedes CHD. However, this does not mean that the relation is definitely causal. Instead, the presence of the relationship in the opposite direction (that is, CHD precedes depression) rather suggests against causality.

The *principle of a dose–response relationship* states that an association is more likely to be causal when there is a dose–response relationship between the variable and the outcome. There is evidence for such a relationship between self-reported depressive symptoms and cardiovascular outcomes: that is, the more severe the (symptoms of) depression, the stronger the association with CHD [29,30]. However, studies comparing the prognostic value of self-reported symptoms of depression with the prognostic value of a diagnosis of MDD report mixed results. Two studies [31,32] found a diagnosis of MDD to be more strongly predictive of cardiovascular outcomes in CHD patients. In contrast, two other studies [33,34] found self-reported symptoms of depression to be a stronger predictor of cardiovascular outcomes in CHD patients. In addition, we recently found that self-reported symptoms of depression were a stronger predictor of poor cardiovascular outcomes than a diagnosis of MDD in a sample of 2,493 MI patients [30]. Finally, in our recent study of 767 MI patients, it was found that an increase of symptoms of depression immediately after an MI predicted cardiac events, whether or not these symptoms reached the level of severity of a clinical depression [35].

Thus, there is evidence for a dose–response relationship between self-reported depressive symptoms and cardiovascular outcomes, which is supportive of the idea of causality. However, a diagnosis of MDD is not necessarily a stronger predictor of cardiovascular outcomes than self-reported depressive symptoms, which seems contradictive. In research, self-report questionnaires are used more often than clinical diagnoses of depression. A characteristic of a Diagnostic Statistical Manual of Mental Disorders (DSM)-IV diagnosis of depression is that by definition, the symptoms of depression cannot be the result of a physical disease or medication use [36]. In contrast, self-report questionnaires do not distinguish between different causes of symptoms of depression. Therefore, it cannot be excluded that depressive symptoms reported on self-reported questionnaires may be an expression of CHD symptoms, which could explain the association with worse cardiovascular prognosis.

The *principle of plausibility* states that causality is more likely when there are plausible theories or mechanisms to explain an association. Indeed, several plausible (mediating) mechanisms have been proposed through which depression can cause poor CHD outcomes. These mechanisms include, among others, elevated inflammation or platelet activation, changes in autonomic nervous system functioning and in hypothalamic-pituitary-adrenal axis functioning [20,37]. They may be causally involved in the association between depression and CHD. On the other hand, instead of forming pathways between depression and CHD, they may also confound the association by underlying the development of both depression and CHD. Biological causes for depression are thought to involve vascular disease, atherosclerosis and systemic inflammation [38], which are processes also strongly involved in cardiac disease. For example, there is evidence that systemic inflammation is involved in the development of both depression and CHD [39-43].

The *principle of experimental manipulation* states that an association is more likely to be causal when manipulation of the determinant variable results in changes in the outcome. This may be the most important principle arguing against causality in the association between depression and CHD. Studies using depression treatment methods recommended by clinical guidelines, in which attempts have been made to improve depression (CREATE [44], SADHART [45,47], ENRICHD [46]) have been moderately effective in doing so, but did not result in subsequent improvement of CHD outcomes. Potentially, the improvements in depression were too small to affect CHD outcomes. On the other hand, it may indicate that depression is not causal of CHD.

In summary, despite decades of research, based on the evidence for criteria of causality discussed above, evidence appears to be against depression as a causal risk factor for CHD. Depression can, therefore, best be conceptualized as a variable risk marker for CHD and its progression [48].

The consequences of this conceptualization of the association between depression and cardiovascular disease are, most importantly, that variations in depression are associated with variations in CHD and cardiovascular outcomes, but that experimental manipulation of depression does not change the CHD outcomes, as shown. Although depression is considered a mental disorder, for some CHD patients, some depressive symptoms may occur as a *physical* response to the cardiac disease. For example, fatigue is a symptom of depression, but may also be a consequence of CHD. Therefore, we propose an alternative theory, based on the fact that depression is highly heterogeneous, and typically, those symptoms and subtypes of depression that are most strongly associated with cardiac prognosis are those that are most likely a physical response to cardiac disease. The following areas of research will be discussed in light of the theory of cardiac disease severity as a confounder in the association between depression and CHD: 1) the cardiotoxicity of somatic/affective depressive symptoms; 2) the cardiotoxicity of treatment-resistant depression; and 3) residual confounding.

## Discussion: depression as a marker of cardiac disease severity

### Somatic/affective vs. cognitive/affective symptoms

There is evidence for two prototypical symptom clusters of depression in CHD patients, consisting of somatic/affective and cognitive/affective symptoms. Somatic/affective symptoms of depression are physical symptoms, such as fatigue, psychomotor changes, changes in appetite and weight, difficulty working, sleeping problems and pain [38]. Cognitive/affective symptoms include symptoms such as depressed mood, loss of interest, suicidal ideation, pessimism, interpersonal sensitivity and feelings of failure, guilt, self-dislike, self-accusation and self-criticism [38]. Due to their somatic nature, somatic/affective symptoms may conceptually show greater overlap with cardiac disease than cognitive/affective depressive symptoms. That is, somatic/affective symptoms may be a direct (fatigue) or indirect (work difficulties) physical consequence of the cardiac disease. If cardiac disease is an important confounder in the association between depression and cardiac prognosis, then particularly somatic/affective depressive symptoms should be related to worse cardiac prognosis and to pathophysiological underlying processes.

Somatic/affective symptoms indeed are more strongly associated with worsened cardiac outcomes than cognitive/affective symptoms [38]. In a study of patients with stable CHD, each somatic symptom of depression was associated with a 14% higher risk of new cardiac events after adjustment for cardiac risk factors, whereas cognitive symptoms of depression were not [49]. In another study, somatic/affective and appetitive symptoms of depression were both associated with, respectively, 35% and 42% increased risk of cardiac mortality and morbidity, but cognitive/affective symptoms were not [50]. In MI patients, somatic/affective symptoms were found to be more strongly associated with cardiac health status (left ventricular ejection fraction (LVEF), Killip class and previous MI) and cardiac prognosis and mortality than cognitive/affective symptoms in several studies [51-53]. Smolderen *et al.* found that somatic symptoms of depression were associated with long-term outcomes in MI patients, but cognitive symptoms of depression were not [54]. Recently, Bekke-Hansen *et al.* found that somatic/affective symptoms at 12 months after an MI predicted all-cause and cardiac mortality, but no such association was found for cognitive/affective symptoms [55]. In contrast, two studies found cognitive/affective depressive symptoms to be more predictive of cardiac outcomes [56,57]. However, these two studies were both performed in coronary artery bypass patients evaluating depressive symptoms postoperatively, whereas all former studies evaluated depressive symptoms in CHD patients (either stable CHD or within several months after an acute cardiac event). One other study in MI patients found three out of four somatic symptoms of depression (fatigue, appetite problems and psychomotor changes), but also two out of five cognitive depressive symptoms (lack of interest and suicidal ideation) to be associated with poor cardiac outcomes [58]. However, this latter study was the only study that assessed depressive symptoms with a diagnostic interview. Thus, in all studies on CHD patients, except those just after coronary artery bypass grafting (CABG) surgery, self-reported somatic/affective symptoms of depression predicted poor cardiac outcomes more than cognitive/affective symptoms. This is suggestive of a specific link between self-reported somatic/affective symptoms and CHD.

Somatic/affective symptoms may be associated more with different underlying mechanisms than cognitive/affective symptoms, resulting in somatic affective symptoms being particularly cardiotoxic [59]. Most studies only find a link between physiological processes and somatic/affective symptoms. One study found that low heart rate variability, which is associated with worsened cardiac outcomes, was associated with somatic/affective symptoms but not with cognitive/affective symptoms of depression in patients with stable CHD [60]. Also, in several studies, somatic/affective symptoms, but not cognitive/affective symptoms, have been associated with atherosclerosis in otherwise healthy people [61,62], and with visceral obesity [63]. In addition, otherwise healthy patients with atypical depression (increased appetite, increased sleep) were found to have higher body mass index and higher risk of metabolic syndrome than patients with melancholic depression [64]. Apparently, somatic/affective, but not cognitive symptoms of depression are associated with biological mechanisms involved in CHD. This link may, therefore, be particularly strong in patients with a recent cardiac event, such as a myocardial infarction. Delisle *et al.*, for example, found that hospitalized depressed MI patients had higher Beck Depression Inventory somatic symptom scores than did depressed psychiatry outpatients [65]. Together, findings on the relationship of somatic/affective symptoms with cardiac prognosis and underlying biological mechanisms suggest that somatic/affective depressive symptoms are a physiological consequence of CHD, which explains at least part of the association between the two.

Cognitive/affective and somatic/affective symptoms often occur together. These two symptom clusters of depression are in fact continuous phenomena, making it difficult to give an exact figure of the prevalence of the two subtypes and their co-occurrence. We believe that there will be continuous transitions between two prototypical forms of depression, while any mixture of cognitive and somatic affective symptoms may develop in a particular individual with CHD [38]. Both clusters may thus be present at the same time, in some cases there may be a sequential pattern of symptoms, and in some a clear predominance of one of the clusters may be present. Future research should further explore these issues in CHD patients. Thus, although both symptom profiles may be present within the same individual, the somatic/affective symptom profile is often found to be associated with worse cardiovascular prognosis, independent of the cognitive/affective symptom profile.

### Treatment-resistant depression and cardiac prognosis

Another subtype of depression that is related to cardiac disease is treatment-resistant depression, which is particularly associated with the risk of poor cardiovascular outcomes [38,66]. In the Montreal Heart Attack Readjustment Trial (M-Hart), the effects of a psychosocial nursing intervention on psychological distress, mortality and new cardiovascular events were evaluated in 1,376 post-MI patients [67]. Patients who showed persisting or worsening psychological distress despite the intervention had an increased risk of dying or of cardiac hospital readmissions within the subsequent year [68]. Milani *et al.* evaluated the effects of a cardiac rehabilitation program with exercise training on depressive symptoms and all-cause mortality in CHD patients. They found patients with persisting or increasing depressive symptoms during the rehabilitation program had higher all-cause mortality rates than patients with decreasing or constantly low levels of depressive symptoms [69]. More recently, this finding was replicated in CHD patients with additional heart failure [70]. The Myocardial Infarction and Depression Intervention Trial (MIND-IT) evaluated the effects of the antidepressants mirtazapine and citalopram on depression and risk of new cardiac events in depressed MI patients. Patients who did not respond to the treatment significantly more often had a new cardiac event (25.6% vs. 7.4%) compared with those who did respond [71]. The Sertraline Antidepressant Heart Attack Trial (SADHART) included depressed acute coronary syndrome patients in a six-month randomized treatment trial of sertraline vs. placebo. Patients with treatment-resistant depression were at increased risk of all-cause mortality up until eight years after treatment initiation, and this increased risk was also found for patients with persisting depression who were treated with placebo [45,47]. Similar results were found in the Enhancing Recovery in Coronary Heart Disease (ENRICHD) trial, which evaluated the effects of six months’ treatment with cognitive behavioral therapy supplemented with sertraline on cardiovascular outcomes and mortality in depressed MI-patients. Patients in whom the depressive symptoms did not improve had increased mortality rates compared to those whose depressive symptoms did improve [72,73]. Recently, the investigators showed that this increased risk was due to the persistence of somatic/affective depressive symptoms, but not to cognitive/affective depressive symptoms [73].

One explanation for the association between treatment-resistant depression and worse cardiac prognosis is that underlying factors relate to both the treatment nonresponse and the poor cardiac prognosis, such as the cardiac disease itself. That is, patients with treatment-resistant depression may have a constantly severe or even deteriorating underlying cardiac disease. A constantly severe or deteriorating heart disease would be reflected in depressive symptoms that persist over time, do not respond to traditional depression treatment, and that are associated with worse cardiovascular prognosis. This is consistent with the hypothesis that depression is a variable risk marker for cardiac outcomes.

### Residual confounding

If depression were a variable risk marker, one would expect the association between depression and cardiac prognosis to be attenuated after adjustment for potential confounders, like the severity of the cardiac disease. Still, the association between depressive symptoms and cardiovascular prognosis remains, even after adjustment for severity of the heart disease and other potential confounders [1,2]. This suggests that depression is an independent risk factor for CHD. Instead of this, we would rather argue that this is the result of incomplete adjustment. When cardiac disease severity is incompletely or imprecisely measured, statistical adjustment for cardiac disease severity may lead to an underestimation of its underlying role. This phenomenon is known as residual confounding [74] (that is, due to imprecise measurement of parameters) or unmeasured confounding (that is, due to unmeasured parameters). A simulation study showed that associations found in observational studies, such as those between depression and cardiac prognosis, can be generated by residual and unmeasured confounding alone [75]. In contrast to observational studies, experimental studies with randomized designs minimize confounding by unmeasured as well as measured factors. If an association is found in an observational study, but not in an experimental study, it is likely that unmeasured or imprecisely measured factors confound the association. This may be the case for depression and cardiac prognosis, as observational studies consistently find an association between depression and cardiac prognosis [1-3], but experimental manipulation of the depression in a randomized trial does not affect cardiac prognosis [45-47,76].

## Conclusion

We suggest that, based on current evidence, depression can best be defined as a non-causal variable risk marker for CHD, and causality cannot be established. In addition, recent literature on the heterogeneity of depression demonstrates that subtypes of depression that are specifically cardiotoxic may be present in some patients. Although we believe that particularly the role of underlying cardiovascular disease processes in explaining the association between (subtypes of) depression and CHD is currently underrated, there are likely additional mechanisms underlying the association. As the association between depression and CHD is complex, and depression is strongly heterogeneous in CHD patients, different mechanisms and combinations of mechanisms are probably involved in different patients.

## Abbreviations

CABG: Coronary artery bypass grafting; CHD: Coronary heart disease; CREATE: Cardiac randomized evaluation of antidepressant and psychotherapy fficacy; ENRICHD: Enhancing recovery in coronary heart disease; LVEF: Left ventricular ejection fraction; MDD: Major depressive disorder; M-HART: Montreal heart attach readjustment trial; MI: Myocardial infarction; MIND-IT: Myocardial infarction and depression intervention trial; SADHART: Sertraline antidepressant heart attack randomized trial.

## Competing interests

The authors declare they have no competing interests.

## Authors’ contributions

AM drafted the manuscript. PdJ conceived of the paper, and critically reviewed and revised the manuscript. MZ assisted in drafting the manuscript and critically reviewed and revised the paper. All authors read and approved the final manuscript.

## Pre-publication history

The pre-publication history for this paper can be accessed here:

http://www.biomedcentral.com/1741-7015/11/130/prepub
